# Fluoride – a scoping review for Nordic Nutrition Recommendations 2023

**DOI:** 10.29219/fnr.v67.10327

**Published:** 2023-12-29

**Authors:** Marian Kjellevold, Maria Kippler

**Affiliations:** 1 Department of Seafood, Nutrition and Environmental State, Institute of Marine Research, Bergen, Norway; 2 Institute of Environmental Medicine, Karolinska Institutet, Stockholm, Sweden

**Keywords:** fluoride, trace elements, caries, fluorosis, nutrition recommendations

## Abstract

Fluoride has a well-documented role in the prevention and treatment of dental caries, but the mechanism is attributed to local effects on the tooth enamel surface rather than systemic effects. Fluoride is not considered essential for humans, no deficiencies are known, and no optimal range, which will not result in moderate fluorosis in some individuals, can be set. Recently, research studies have shown evidence for a relationship between fluoride intake and cognitive outcomes and interaction with iodine nutrition, but the evidence is weak so more data are warranted. For performing longitudinal cohort studies in the Nordic and Baltic region, data on fluoride in food and beverages need to be implemented in food composition tables. As the preventive effects of fluoride are mainly from topical treatment, monitoring of fluoride intake and establishing reference values for fluoride in urine and plasma are warranted to establish safe intake values.

## Popular scientific summary

- Fluoride is a trace element with well-established effects on dental caries prevention.
- Drinking water is generally the dominant source of fluoride.
- Data on fluoride levels in foods are sparse.
- Fluoride deficiency has not been reported in humans, and fluroide is not an essential element.
- Low-fluoride exposure may have negative effects on iodine status and cognitive development, but evidence is limited.

Fluoride is the 13^th^ most abundant element in the earth’s crust and is, consequently, an inevitable part of the biosphere and human life. Water is generally regarded to be the most important dietary fluoride source, while few data exist on fluoride levels in food. Surface water is generally low in fluoride, while groundwater may contain high concentrations depending on, for example, geological processes. In some areas of the world, fluoride is added to domestic water sources, salt or milk. A recent Cochrane systematic review concluded that there is low-quality evidence for that fluoridated milk contributed to reduction in caries (1). In 1974, the World Health Organization Expert Committee concluded that water fluoridation is safe and cost-efficient and should normally be in the range 0.5–1.0 mg F/L (2). Fluoridation of domestic water was discussed in Norway and Sweden in the 1950s but was not implemented (3). In the Nordic and Baltic areas, fluoride concentrations in drinking water varies, and some water sources are naturally higher than the guideline value of 1.5 mg F/L. A variety of fluoride supplements are advocated in order to prevent dental caries, and mild dental fluorosis has been accepted as a side effect due to caries-protective benefits (4). Even with very low-fluoride intake from water, a certain level of dental fluorosis will be found, and thus, there exists no critical or optimal level for which the effect on the dental enamel will not be manifested (5). Based on current evidence, it is difficult to conclude on an ‘optimal’ range for fluoride intake at the individual level, but it can be used at the population level to guide programs on community fluoridation (6). Since the last update of the Nordic Nutrition Recommendations (NNR2012), emerging evidence on adverse health outcomes of low-to-moderate fluoride exposure has become available.

The aim of this scoping review is to describe the totality of evidence for the role of fluoride for health-related outcomes as a basis for setting and updating dietary reference values (DRVs) for the Nordic Nutrition Recommendations (NNR) 2023 (Box 1).

Box 1 Background papers for Nordic Nutrition Recommendations 2023

- This paper is one of many scoping reviews commissioned as part of the Nordic Nutrition Recommendations 2023 (NNR2023) project (7)
- The papers are included in the extended NNR2023 report but, for transparency, these scoping reviews are also published in Food & Nutrition Research
- The scoping reviews have been peer reviewed by independent experts in the research field according to the standard procedures of the journal
- The scoping reviews have also been subjected to public consultations (see report to be published by the NNR2023 project)
- The NNR2023 committee has served as the editorial board
- While these papers are a main fundament, the NNR2023 committee has the sole responsibility for setting dietary reference values in the NNR2023 project

## Methods

For fluoride, no *de novo* systematic review or qualified systemic review was available (8). Thus, a systematic literature search was performed in PubMed/MEDLINE in accordance with the NNR2023 project (9), using the following search string: *“fluoride”[MeSH Terms] AND (“2011”[PDAT] : “3000”[PDAT]) AND review[Publication Type] AND Humans[Filter]*.

This search resulted in 494 records published between January 2011 and June 6^th^, 2022, of which 13 were read in full text (Table 1). The selection was done in accordance with the eligibility criteria described in Christensen et al. (10). In addition, the following sources were included as source of evidence: the European Food Safety Authority’s (EFSA) *Scientific Opinion on Dietary Reference Values for fluoride* (10) and the Norwegian Scientific Committee for Food and Environment’s (VKM) *Assessment of dietary intake of fluoride and maximum limits for fluoride in supplements* (11), in addition to key references found in articles and sources mentioned earlier.

**Table 1:** Synthesis of results from search on new scientific data that possibly may argue for changes in existing DRVs or FBDGs relevant for fluoride.

No	Exposure	Outcome	Title	Comments and considerations	Reference
1	Fluoride	Iodine deficiency	Fluoride Exposure Induces Inhibition of Sodium/Iodide Symporter (NIS)Contributing to Impaired Iodine Absorption and Iodine Deficiency: MolecularMechanisms of Inhibition and Implications for Public Health	- Suggests that high F exposure may affect iodine uptake and bioavailability - Review, but no systematic approach or meta-analysis	(16)
2	Fluoride	Degenerative eye disease	The Contribution of Fluoride to the Pathogenesis of Eye Diseases: Molecular Mechanisms and Implications for Public Health	- Suggests that high F exposure leads to degenerative eye disease - Review, but no systematic approach or meta-analysis	(17)
3	Fluoride	Alzheimers disease	Potential Role of Fluoride in the Etiopathogenesis of Alzheimer’s Disease	- Review, but no systematic approach or meta-analysis	(18)
4	Fluoride	Dental caries and fluorosis	Understanding Optimum Fluoride Intake from Population-Level Evidence	- Good discussion about DRVs for F - Review, but no systematic approach or meta-analysis	(19)
5	Fluoride	Dental caries and fluorosis	Current Guidance for Fluoride Intake: Is It Appropriate?	- Good discussion about DRVs for F - Review, but no systematic approach or meta-analysis	(20)
6	Fluoride	Dental caries and fluorosis	Review of Fluoride Intake and Appropriateness of Current Guidelines	- Good discussion about DRVs for F - Review, but no systematic approach or meta-analysis	(6)
7	Water fluoride	Intelligence (IQ)	Association between water fluoride and the level of children’s intelligence: adose-response meta-analysis	- 26 studies - 7258 children - Most studies from before NNR2012, some after- Newer studies mostly from India, China and Iran; it is somewhat unclear if this is generalizable to the Nordic/Baltic setting - Higher F associated with lower IQ- Dose-response present - Robust in sensitivity analyses	(21)
8	Fluoride in milk	Dental caries	Fluoridated milk for preventing dental caries	- Cochrane review of milk as a vector for F intake to prevent caries - Conclusion: low RCT evidence of effect	(1)
9	Fluoride in drinking water	Hip fracture	Exposure to fluoride in drinking water and hip 10 fracture risk: a meta-an11alysis of observational studies	- 14 observational studies - Cohorts from US, Europe and Asia - Only one of the 14 included studies was published after NNR2012 - No association between F and fracture - Seems like high quality work- Substantial heterogeneity	(13)
10	Fluoride in drinking water and well water	- Neuro-developmental outcomes - IQ scores - Cognitive function	Developmental fluoride neurotoxicity: a systematic review and meta-analysis	- 27 epi studies - Studies mainly from China - Inverse association between F and various neurodevelopmental outcomes - Robust in sensitivity analyses	(14)
11	Fluoride in drinking water, coal burning and/or urinary F	- Neurotoxicity - Intellectual disability - IQ scores	Developmental fluoride neurotoxicity: an updated review.	- Cross-sectional studies (12), prospective studies (5) - Suggests that elevated F intake can result in IQ deficits - Review, but no systematic approach or meta-analysis	(15)
12	Fluoride in drinking water and/or urinary F	- Neurotoxicity - IQ scores - school performance	Toxicity of fluoride: critical evaluation of evidence for human developmental neurotoxicity in epidemiological studies, animal experiments and in vitro analyses.	- 21 of 23 studies report association between high F exposure and reduced IQ - Low quality studies - Does not support F as neurotoxicant at the current exposure levels in Europe - Review, but no systematic approach or meta-analysis	(22)
13	Fluoride in drinking water	- IQ - Mental abilities	Fluorosis and cognitive development among children (6–14 years of age) in the endemic areas of the world: a review and critical analysis.	- 57 studies - 7 found no effect, 50 (China, India, Iran, Mexico) found relation between F and neuronal impact	(23)

## Physiology and metabolism

Fluoride in drinking water is effectively absorbed (>90%), but complex-bound fluoride in foods is less well absorbed. The bioavailability of fluoride from food varies from 2 to 79% and depends on factors like pH and mineral content of the food. Fluoride from bone meal, fish bone meal, canned sardines, and chicken bone meals has been shown to have low availability (4–24%), most likely due to high calcium content (12, 24). Approximately 50% of the absorbed fluoride is excreted via the kidneys, and the rest is incorporated into the bones and, in childhood, into the teeth. Thus, the main proportion of fluoride in the body is complex-bound to calcium in the skeleton and tooth tissues. These fluoride complexes can replace the hydroxyl ions in hydroxyapatite crystals, making the crystals less acid-soluble. Fluoride has anticariogenic and antimicrobial properties due to acidification of the bacterial cytoplasm and disruption of the bacterial metabolism (25). Fluoride increases bone density by stimulating bone formation, but the risk of bone fractures increases with long-term excessive intake (10, 11).

## Assessment of nutrient status

Dental and skeletal fluorosis are the adverse effects of excessive fluoride ingestion best described in the literature. However, the manifestation of fluorosis is first visible several years after exposure when it is too late for interventions. The World Health Organization (WHO) has categorized bone and teeth as historical markers, nails and hair as recent markers, and urine, plasma, and saliva as contemporary markers (2). However, there is no consensus regarding which is the most suitable biomarker. Nails and hair have been shown as promising biomarkers for monitoring systemic fluoride levels from exposure through drinking water (26). Salivary biomarkers have been identified as promising but cannot be used alone, and more research is required (27). Total fluoride in urine and nails is found to correlate with the severity of dental fluorosis, and regarded adequate for assessing relationships in fluoride endemic areas (28). Urine has also been shown to be a promising biomarker on the population level, but not on an individual level (2). Therefore, a biomarker suitable for assessing fluoride exposure in individuals in non-endemic areas is currently lacking.

## Dietary sources and intake

In the most recent EFSA report, they conclude that no data are available for Europe due to the lack of fluoride values in food composition tables (29). However, since the EFSA report was published, the Danish food composition table (2022) has included some data on fluoride (approximately 70 food entries). The concentrations reported are low, ranging from 0 to 0.2 mg F/kg (30). This is in accordance with the general literature. Fluoride levels in foods are generally low, with a few exceptions like seafood and tea (brewed on leaves from the plant *C. sinensis*). In a study from 1981, mean content of fluoride in various seafood was 0.71 mg/kg, ranging from 0.2 to 2.0 (31). A recent study from Denmark shows that daily consumption of tea by pregnant women exceeds safe limits of fluoride intake (32). Fish eaten with the bones, such as canned sardines, some teas and mineral waters, and drinking water in some areas have the highest content. Foods prepared with fluoridated water (e.g. soup and infant formula) may be relevant fluoride sources, and in high-fluoride areas, food has been shown to provide a major contribution to the fluoride intake in children (33). Little data on fluoride intake from food are available, but according to EFSA (29), fluoride intakes from food (except for fruit juice, mineral water, and tea) for young children, older children, and adults are reported to be 0.04, 0.11, and 0.12 mg/day, respectively. Fruit juice, mineral water, and tea are estimated to contribute with 0.01 mg, 0.06 mg, and 0.26 mg of fluoride, respectively.

Drinking water is generally the dominant source of fluoride. Tap water and other water sources such as mineral waters can contribute to various amounts of fluoride depending on the concentration in the water. Surface water usually has a low fluoride concentration (below 0.5 mg/L). Ground water concentration in EU is generally low, but there are large regional differences. In Sweden, fluoride concentrations in drinking water from water treatment plants have been reported to mostly range between 0 and 1.5 mg/L, with a maximum level of 4.1 mg/L (34), while in private wells in South-Eastern Sweden, it was found that 24% out of about 4,800 wells exceed 1.5 mg/L (35). In Norway, a study from 2017 found that 4 of 201 waterworks had fluoride concentrations exceeding guideline value of 1.5 mg F/L (36). In a study from Western Norway, the fluoride concentration in selected wells ranged from 0.51 to 8.0 mg F/L, and drinking water was the only dietary variable associated with increased risk of dental fluorosis (37). In Denmark, analyses of drinking water show most sources being low and below 1.5 mg F/L (38). In the EFSA report, the contribution of fluoride from water and water-based beverages in adolescents (>15 years) and adults was reported to be around 0.13 mg/day at a concentration of 0.1 mg F/L and about 3.02 mg/day at a concentration of 0.8 mg F/L, 5.66 mg/day at a concentration of 1.5 mg F/L and 8.40 mg/day at a concentration of 3.0 mg F/L (39). Toothpaste can also contribute to the ingested fluoride, especially in small children. It is estimated that in adults, <10% of the toothpaste is ingested because the spitting reflex is well developed, but the intake in children has been reported to be as high as 48% in 2- to 3-year-old, 42% in 4-year-old, 34% in 5-year-old, and 25% in 6-year-old children. In children aged 8–12 years, the ingestion is reported to be ~10% (40). According to VKM, toothpaste and dental fluoride tablets contribute 21% to total intake in 2-year-old children, and 32 and 23% in 4- and 9-year-old children, respectively (11). However, in these estimations, data on fluoride intake from dietary sources other than beverages are missing and are included in ‘all other sources’, which is set to 0.2 mg/day regardless of age.

The EFSA panel recommends systematic collection of analytical data on fluoride in foods (29). This is in accordance with the VKM report, which mentions lack of data on teas on the Norwegian market as one of several uncertainties (11). Recently, foods from the ocean and increased consumption of low-trophic aquatic foods have been suggested as one solution for increased food production (41, 42). Some low-trophic species, like krill, have been shown to contain high-fluoride concentrations (43). Thus, monitoring of fluoride in low-trophic aquatic species is also relevant.

## Health outcomes relevant for Nordic and Baltic countries

### Deficiencies

Fluoride is not regarded to be essential to humans, and caries is not regarded as a result of fluoride deficiency. Thus, deficiency of fluoride will not be discussed further.

### Toxicity

An intake of 2.2 g/kg bodyweight is lethal in adults. In children, 15 mg/kg bodyweight is lethal, and 5 mg/kg bodyweight causes acute symptoms, such as nausea, stomach pain, and vomiting. It is well established that chronic high intakes of fluoride via drinking water can affect skeletal mineralization (10). The most common side effect of high-fluoride intake is dental fluorosis or ‘mottled teeth’. Fluoride can affect bone cell function, bone structure, and bone strength. Several studies have reported that chronic excessive intake of fluoride has been associated with skeletal fluorosis (joint stiffness and skeletal deformities) and also with reduced bone strength and increased risk of fractures (10). On the other hand, observational studies of chronic low-to-moderate fluoride intake and bone health have reported varying results. In a meta-analysis, including 14 studies, exposure to fluoride via drinking water was not associated with the incidence of hip fractures (13). Likewise, in a Swedish cohort study, chronic estimated individual drinking water fluoride exposure was not associated with hip fractures (44). However, in another Swedish cohort of postmenopausal women, low-to-moderate fluoride intake (mean urinary fluoride concentration of 1.2 mg/g creatinine and dietary intake of 2.2 mg/day) was positively associated with the incidence of hip fractures (45). Thus, more prospective studies of low-to-moderate fluoride exposure and bone health are warranted. Human and experimental animal studies have found that high-fluoride intake or exposure may be associated with changes in thyroid hormone levels, such as triiodothyronine, thyroxine, and thyroid-stimulating hormone (TSH) (46, 47), and that the relationship between fluoride and TSH may be modified by iodine status (47). In 2012, a systemic review and meta-analysis, including 27 epidemiological studies, found that children in ‘high-fluoride’ areas had lower IQ scores than those who lived in ‘low-fluoride’ areas (14). Later on, several cross-sectional studies and a few prospective studies from Mexico and Canada with individual exposure data have reported that prenatal and perinatal exposure to fluoride may be negatively associated with cognitive development (15). Nevertheless, more prospective studies are needed, and European data are lacking.

### Upper intake levels and toxicity

The EFSA (48) considered that a daily intake of up to 0.1 mg of fluoride per kg bodyweight in children up to 8 years old leads to no significant occurrence of ‘moderate’ forms of fluorosis in permanent teeth. Based on this, an upper intake level (UL) was set to 1.5 mg/day for children 1–3 years old and 2.5 mg/day for children 4–8 years old. For children 9–14 years old (UL 5 mg/day) and adults (UL 7 mg/day), the UL is based on data from studies on fractures.

## Requirement and recommended intake

In the 2012 NNR, no recommendation for daily fluoride intake was given because it was not considered an essential trace element (49). Since then, no updates have been performed by the US National Academies of Sciences, Engineering, and Medicine (NASEM), while EFSA published a value for adequate intake (AI) in 2013. The U.S. Institute of Medicine (present NASEM) concluded that they were unable to establish an estimated average intake (EAR) due to the absence of data, but set an AI for fluoride that is based on the observed estimated intake judged to reduce the incidence of dental caries in a group of healthy adults (50). For adults, this level was set to 3 mg/day and 4 mg/day for women and men, respectively. For infants and children (>6 months), the AI was set to 0.05 mg F/kg/bw/day (50). The EFSA Panel concludes that the AI of fluoride can be set to 0.05 mg/kg bw/day for both children and adults (29). The main challenge for setting recommended intake in the Nordic and Baltic countries is the lack of food composition data reporting fluoride content in foods, and lack of data on fluoride status in the population. Most of the literatures available are from areas with endemic fluorosis or from areas with generally higher fluoride content in drinking water. Even though there is no strong evidence yet, due to both methodological challenges and few data, the new evidence on the negative effects of low exposure of fluoride on iodine nutrition and cognitive development calls for more studies in low-fluoride areas, inclusion of fluoride in food composition tables, and establishment of reference values for urinary fluoride.
